# Consumption of a soy drink has no effect on cognitive function but may alleviate vasomotor symptoms in post-menopausal women; a randomised trial

**DOI:** 10.1007/s00394-019-01942-5

**Published:** 2019-03-12

**Authors:** Orlaith N. Furlong, Heather J. Parr, Stephanie J. Hodge, Mary M. Slevin, Ellen E. Simpson, Emeir M. McSorley, Jacqueline M. McCormack, Pamela J. Magee

**Affiliations:** 1 Nutrition Innovation Centre for Food and Health, Ulster University, Coleraine, BT52 1SA Northern Ireland UK; 2 Psychology Research Institute, Ulster University, Coleraine, BT52 1SA Northern Ireland UK; 3 Present Address: University of East Anglia, Norwich, UK; 4 Present Address: Clinical Health and Nutrition Centre, Institute of Technology, Sligo, Ireland

**Keywords:** Isoflavones, CANTAB, Menopausal symptoms, Hot flush, Hot flash, Equol

## Abstract

**Purpose:**

Cognitive decline is commonly reported during the menopausal transition, with memory and attention being particularly affected. The aim of this study was to investigate the effects of a commercially available soy drink on cognitive function and menopausal symptoms in post-menopausal women.

**Methods:**

101 post-menopausal women, aged 44–63 years, were randomly assigned to consume a volume of soy drink providing a low (10 mg/day; control group), medium (35 mg/day), or high (60 mg/day) dose of isoflavones for 12 weeks. Cognitive function (spatial working memory, spatial span, pattern recognition memory, 5-choice reaction time, and match to sample visual search) was assessed using CANTAB pre- and post-the 12 week intervention. Menopausal symptoms were assessed using Greene’s Climacteric Scale.

**Results:**

No significant differences were observed between the groups for any of the cognitive function outcomes measured. Soy drink consumption had no effect on menopausal symptoms overall; however, when women were stratified according to the severity of vasomotor symptoms (VMS) at baseline, women with more severe symptoms at baseline in the medium group had a significant reduction (*P* = 0.001) in VMS post-intervention (mean change from baseline score: − 2.15 ± 1.73) in comparison to those with less severe VMS (mean change from baseline score: 0.06 ± 1.21).

**Conclusions:**

Soy drink consumption had no effect on cognitive function in post-menopausal women. Consumption of ~ 350 ml/day (35 mg IFs) for 12 weeks significantly reduced VMS in those with more severe symptoms at baseline. This finding is clinically relevant as soy drinks may provide an alternative, natural, treatment for alleviating VMS, highly prevalent among western women.

## Introduction

Cognitive decline is commonly reported by peri- and post-menopausal women and deteriorations in memory; attention and processing speed have been observed during the menopausal transition [1–4]. These effects have been attributed to a reduction in circulating estrogen concentrations [5]; albeit, this has been contested [6]. Hormone therapy (HT) remains the most effective treatment for vasomotor menopausal symptoms (VMS) [7] and early observational studies supported a beneficial effect of HT on cognitive function [8]. Two recent intervention studies have demonstrated that HT initiated early in menopause has neither beneficial nor harmful effects on cognition [9, 10]. Furthermore, despite HT being commonly prescribed for otherwise healthy young/early post-menopausal women, many refrain from HT use due to the health risks associated with HT use in older women [7, 11]. Alternatively, natural approaches for the treatment and prevention of menopausal symptoms are thus being sought.

Dietary soy isoflavones (IFs) have been reported to be efficacious in the treatment of hot flushes [12] and to have the potential to enhance cognitive function in post-menopausal women, having the ability to cross the blood brain barrier in small amounts [13, 14]. Genistein and daidzein, the main IFs present in the soy bean, can bind to estrogen receptors (ERs), and are classified as selective ER modulators, having a higher affinity for ER-β than ER-α [15, 16]. ERs are localised throughout the adult brain including the hippocampus, where ERβ is more highly expressed in comparison to ERα [17], and are also localised within the prefrontal cortex [5]. These areas are important for learning, memory, attention, and higher order cognitive function, and are particularly susceptible to age-related decline [18]. Both ER-mediated and non-ER-mediated neuroprotective effects have been demonstrated for soy/IFs using in vitro and animal models [19, 20]. Such mechanisms include reduced neuronal loss, inhibition of β-amyloid-induced cell death, facilitation of cholinergic transmission, reduced free radical generation, anti-inflammatory and antioxidant effects [19, 21], and via the modulation of mitochondrial function [20]. Furthermore, a high soy diet [22] and soy isoflavone supplementation [23] has been shown to improve cognitive function in men. Nevertheless, randomised-controlled trials investigating the effects of soy/IFs on cognitive function in post-menopausal women have yielded inconsistent findings, with some showing positive effects on cognitive function [24–29] and others demonstrating null/negative effects [30–33]. Two recent comprehensive reviews [19, 34] have highlighted several variations in study design that have likely contributed to these disparate outcomes including: age; time since menopause; habitual soy/IF intake; IF dose, duration, bioavailability and metabolism, and cognitive function assessment. To date, only one study has investigated the effects of soy/IFs on cognitive function in post-menopausal women using a food (soy milk) [31] rather than a supplement. There is, therefore, a need for additional, well-designed studies to further assess the effects of IFs on cognitive function.

In line with the ‘critical window hypothesis’ of HT and cognitive function that postulates that optimal effects are evident with the early initiation [35], limited evidence suggests that younger post-menopausal women (< 60 years) may gain more cognitive benefit from soy IFs in comparison to their older counterparts [25]. It has also been postulated that the effects of soy on health may be determined by an individual’s equol producer status [36]. S-equol, a potent ligand for ERβ, is an isoflavan formed exclusively via the bacterial conversion of daidzein in the intestine [37] and is only produced by ~ 30% of western populations following a soy challenge [38–40]. The ability to produce equol has been associated with reduced VMS [41]. Furthermore, S-equol supplementation alleviates hot flushes [42] and S-equol derivatives of soy IFs are now recommended by the North American Menopause Society for the non-hormonal management of VMS [43], who have highlighted the need for further studies in this area.

The aim of this study was to investigate the effects of a commercially available soy drink on cognitive function and menopausal symptoms in post-menopausal women within 7 years postmenopause. A secondary aim was to assess the relationship between equol producer status and cognitive function and menopausal symptoms.

## Subjects and methods

### Design

This 12-week parallel group, randomised, controlled trial was conducted between October 2015 and May 2018. All procedures were approved by Ulster University’s Research Ethics Committee (REC/15/0025) and the study was registered at http://www.clinicaltrials.gov (NCT03561662). Participants were recruited throughout the province of Ulster and screening, baseline and post-intervention appointments were conducted before and after the 12-week intervention either at the university, the participant’s home, or at a location convenient for the participant. The duration of intervention was based on previous intervention studies in post-menopausal women that have demonstrated that soy IF supplementation (60 mg/day) for 6 weeks significantly improves frontal lobe function [26], with significant improvements in sustained attention and long-term episodic memory additionally observed when supplementation is extended to 12 weeks [24]. The primary outcome of the study was effect on cognitive function and the secondary outcome was effect on menopausal symptoms. Sub-analysis investigated the effect of the intervention based on severity of VMS at baseline and also investigated cognitive function and menopausal symptoms according to equol producer status.

### Participants

Eligible participants were apparently healthy women within 7 year postmenopause (i.e., 1–7 years since last menstrual period). Post-menopausal status was confirmed based on a serum concentration of follicle-stimulating hormone (FSH) > 30 mIU/ml (assessed via electrochemiluminescence immunoassay on a Cobas 8000 analyzer [Cobas 602 module], Roche Diagnostics at Antrim Area hospital). Exclusion criteria included: surgically induced menopause; habitual consumers of soya foods (> 2 serves/week); current use of HT or IF supplements; antibiotics use within the previous 3 months; current use of psychoactive medication; presence or history of cardiovascular disease, cancer, diabetes, thyroid, renal or kidney disease, alcohol or drug abuse; cognitive impairment as determined by a Mini-Mental State Examination score < 24; psychiatric distress as determined using a General Health Questionnaire-28 [44] score of ≥ 26; red–green colour blind, assessed via the Ishihara test (as CANTAB testing requires colour recognition); abnormal full blood profile (assessed via a Sysmex KX21-N, Sysmex Ltd, UK at Ulster University); and/or insufficient renal/hepatic performance assessed via kidney and liver function tests (assessed photometrically via a Cobas 6000 analyzer [Cobas C501 module], Roche Diagnostics at Causeway Hospital).

### Intervention

An independent clinical trial manager used MINIM software [45] to randomise recruited women to one of the three treatment groups with an allocation ratio of 1:1:1. Participants were asked to consume soy drinks (Alpro^®^) providing a low (10 mg), medium (35 mg), or high (60 mg) dose of IFs daily for a period of 12 weeks and could choose from different flavours of drink (original, unsweetened, chocolate, or strawberry). Limited evidence suggests that IFs consumed in divided doses may be more effective in alleviating menopausal symptoms than a single dose [46, 47], and thus, women were advised to spread their intake throughout the day. The group that consumed the lowest dose of IFs was considered a low-dose control group as beneficial effects of soy IFs on post-menopausal health have previously been observed in intervention studies at much higher doses [48]; furthermore, it was not possible to obtain a placebo control drink. The previous soy/IF intervention studies on cognitive function in post-menopausal women have used doses ranging from 60 to 160 mg IFs per day [24–33]. This study utilised a dose that was achievable in a commercially available soy drink and at a volume that was easily incorporated into an individual’s daily diet. Compliance was monitored by measuring plasma concentrations of soy IFs. Total genistein, daidzein, and equol concentrations were assessed using LC–MS/MS by LGC Limited (Cambridgeshire, UK). Equol producers were defined as those with a plasma equol concentration of > 20 nmol/l (5 µg/l) [40].

### Dietary intake, anthropometrics, and general health and lifestyle

Weight (kg) and height (cm) were measured at baseline and used to calculate BMI [weight (kg)/height (m)^2^]. Body weight was measured to the nearest 0.1 kg using Seca 770 electronic weighing scales (Brosch Direct Ltd, Peterborough, United Kingdom), without footwear and heavy clothing. Standing body height was measured to the nearest 0.1 cm using a Seca 220 stadiometer (Seca Ltd, Hamburg, Germany). The participant stood without footwear, with their heels together, hands and arms hanging relaxed, and measurements were taken with the Frankfurt plane in a horizontal position. Dietary intake was assessed at baseline and post-intervention using a 4-day food diary. Participants received instructions on how to complete the diary from a trained researcher and dietary intake was analysed using Nutritics nutritional analysis software [49]. A general health and lifestyle questionnaire were completed by participants at baseline and provided information on age, gender, marital status, education level, occupation, smoking habits, alcohol use, dietary habits, and physical activity.

### Blood collection and processing

Fasted blood samples were collected by a trained phlebotomist before and after intervention for the analysis of serum FSH and plasma IF concentrations. Participants were instructed to fast from 10 pm the night prior to blood sampling and water intake was encouraged. Fasted blood samples were obtained from the antecubital fossa using a 21-gauge butterfly needle and 8 ml serum and 9 ml ethylenediametetraacetic acid (EDTA) plasma vacutainer tubes (Greiner Bio-One GmbH, Kremsmunster, Austria). Following inversion, serum samples were allowed to clot for > 60 min and plasma samples placed in refrigeration until full blood profile analysis. Following this, all tubes were centrifuged at 2200 rpm for 15 min at 4 °C to allow the separation of whole blood into its respective components. Following separation, serum and plasma samples were divided into aliquots and stored at − 80 °C until further analysis.

### Cognitive function

Pre- and post-intervention cognitive function was assessed in the morning, after participants had consumed a standard, caffeine-free, breakfast. Cognitive function was assessed using the Cambridge Neuropsychological Test Automated Battery (CANTAB Research Suite; Cambridge Cognition, UK) [50]. CANTAB has been extensively validated for assessing brain-to-behaviour relationships in adult populations [51, 52], has proven test–retest reliability [53], and is deemed suitable for use with older adults [51]. The following tests were used: spatial working memory (SWM), spatial span (SSP), pattern recognition memory (PRM), 5-choice reaction time (RTI), and match to sample visual search (MTS). The tests chosen activate areas of the brain that are associated with cognitive decline during the menopausal transition and that are sensitive to hormonal changes, including the hippocampus [54] and prefrontal cortex [5]. SWM and SSP activate the temporal and frontal lobe regions of the brain; PRM activates the temporal lobe, hippocampus and amygdala; RTI and MTS activate the fronto-striatal circuitry [55]. The procedure for assessing SSP, RTI, and MTS is described in detail elsewhere [56]. Spatial working memory (SWM), a sensitive measure of frontal lobe and executive function, requires retention and manipulation of visuospatial information. The test began with four coloured squares (boxes) shown on the screen. Participants were required, by selecting boxes and using a process of elimination, to find one blue ‘token’ in each of four boxes (only one token is hidden at a time) and use them to fill up an empty column on the right-hand side of the screen. Touching any box in which a token has already been found is an error. The trial was then repeated three times with four boxes and then progressed to four trials with six boxes and four trials with eight boxes. The colour and position of the boxes used are changed from trial to trial to discourage the use of stereotyped search strategies and a practice test was completed prior to testing. The outcome measure was SWM total errors, i.e., the number of times which a box is selected that is certain not to contain a token and, therefore, should have not been visited by the participant.

Visual memory was assessed using the Pattern Recognition Memory (PRM) test in a two-choice forced discrimination paradigm. Participants were asked to remember a series of 12 abstract-coloured patterns; each presented for 3 s. They were then presented with a series of 12 pairs of old–new patterns and were asked to touch the pattern seen previously in each case. This procedure was repeated with a second set of 12 patterns followed by 12 pairs of patterns for recognition. The outcome measure was mean correct latency, i.e., the mean time (milliseconds) to respond correctly.

### Menopausal symptoms

The Greene Climacteric Scale [57] was used to assess menopausal symptoms at baseline and post-intervention. This 21-item scale provides three main independent measures of psychological, somatic, and vasomotor symptoms. Participants were asked to indicate the extent to which they were currently bothered by the list of symptoms on a scale from 1 ‘not at all’ up to 4 ‘extremely’.

### Statistical analyses

An a priori power calculation was conducted using spatial working memory total error data obtained from the study of Thompson et al. [58]. Based on the probability of a type 1 error (*α*) = 0.05 and a Power of 0.9, 41 participants were required in each group to be able to reject the null hypothesis that the population means of the treatment groups are equal. To allow for dropouts, we aimed to recruit 150 women to the study. All statistical analyses were performed using the Statistical Package for the Social Sciences (SPSS) with significance set at *P* < 0.05 throughout (IBM SPSS Statistics for Windows, version 24.0, IBM Corp, Armok NY). Only those participants that had completed cognitive testing at both baseline and post-intervention were included in the analysis. Intention-to-treat analysis was also performed including all participants randomised at baseline and did not change the primary outcome findings. The Shapiro–Wilk test was used to determine whether data followed a normal distribution and skewed variables were log-transformed to attain a normal distribution prior to analysis or analysed using non-parametric statistical tests. Transformations were applied to SWM, SSP, PRM, RTI, and MTS data. Descriptive statistics were used to present participant characteristics at baseline. The effect of intervention on the primary outcome measures of cognitive function (SWM, SSP, PRM, RTI, and MTS) was analysed using an analysis of covariance (ANCOVA) with baseline measures included as covariates. The secondary outcome measure of menopausal symptoms was analysed using a Kruskal–Wallis test. IF concentrations were compared between groups using a Kruskal–Wallis test with post hoc analysis conducted using a Mann–Whitney *U* test. Two outliers with post-intervention genistein concentrations of > 800 ng/ml in the low-dose treatment group and one in the high-dose group (baseline genistein of 275 ng/ml) were removed prior to statistical analysis of IF concentrations between groups. Sub-analysis was conducted to determine if the effect of the intervention on cognitive function and menopausal symptoms was significantly different between equol producers and non-producers using a Mann–Whitney *U* test. This test and one-way ANOVA with quadratic contrasts were also used in sub-analysis to investigate the effect of the intervention on VMS stratifying women according to severity of VMS at baseline. Dietary intake pre- and post-intervention was analysed using the Wilcoxin Signed-Rank test.

## Results

**Fig. 1:**
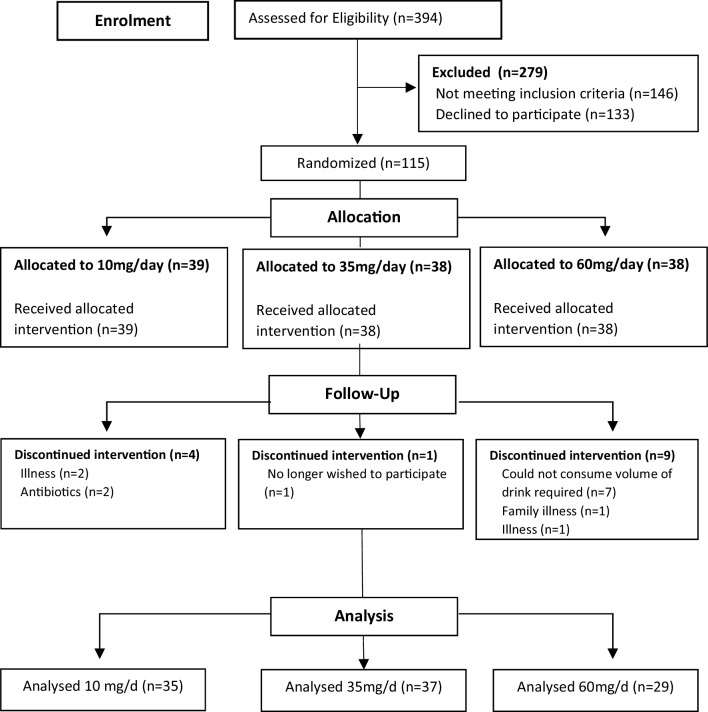
CONSORT diagram of participant flow. A total of 394 women were assessed for eligibility with 279 excluded due to not meeting the inclusion criteria (*n* = 146) or not wishing to participate in the study (*n* = 133). Remaining post-menopausal women were randomised to receive a soy drink (Alpro^®^) and asked to consume a volume providing either 10 mg (*n* = 39), 35 mg (*n* = 38) or 60 mg (*n* = 38) IFs daily. A total of 14 participants were lost to follow up owing to illness (*n* = 3) unrelated to the intervention, antibiotic use (*n* = 2), no longer wishing to participate (*n* = 1), family illness (*n* = 1) or being unable to consume the soy drink (*n* = 7). A total of 101 women completed the study and were included in the final analysis

A total of 101 post-menopausal women completed the study and were included in the final analysis. Participant progress through the study is illustrated in the CONSORT diagram [59] in Fig. 1. Baseline demographic characteristics of the study participants are shown in Table 1. There was no significant difference at baseline between the groups for any of the characteristics presented. Table 2 shows the effect of the intervention on cognitive function; no significant differences were observed between the groups for any of the cognitive function outcomes measured (RTI, SSP, SWM, PRM, and MTS). The soy drink had no effect on menopausal symptoms overall (Table 3); however, when women were stratified according to the severity of their VMS (hot flushes and night sweats) at baseline by splitting into two groups above/below mean, women with more severe VMS at baseline in the medium group had a significant reduction in symptoms after consuming the soy drink for 12 weeks, in comparison to those with less severe symptoms at baseline (Table 4). There was a significant quadratic trend for dose (*P* = 0.011) with the observed reduction in symptoms in the medium-dose group being significantly greater in comparison to that observed in women with more severe VMS at baseline in the low-dose control group (*P* = 0.018) and the high-dose group (*P* = 0.046).

**Table 1:** Baseline participant characteristics

Measure^a^	Soy IF treatment group		
	Low (*n* = 35)	Medium (*n* = 37)	High (*n* = 29)
Age (years)	53.69 ± 3.72^b^	53.86 ± 3.28	53.72 ± 4.62
Height (m)	1.61 ± 0.065	1.64 ± 0.062	1.63 ± 0.073
Weight (kg)	70.32 ± 12.36	71.48 ± 13.85	72.91 ± 14.86
BMI (kg/m^2^)	26.97 ± 5.22	26.76 ± 5.73	27.37 ± 5.77
Non-smokers (%)	94.12	100	100
Alcohol (units/week)	7.64 ± 6.58	11.73 ± 12.60	7.44 ± 10.21
Education level (*n*)			
Primary	0	1	0
Secondary	19	13	12
Tertiary	15	21	16
PA (METs/week)	165.17 ± 56.32	184.70 ± 57.48	192.74 ± 76.98
LMP (months)	31.97 ± 19.05	39.33 ± 21.99	39.38 ± 23.25
FSH (IU/ml)	83.02 ± 34.44	88.79 ± 28.33	85.84 ± 33.84
GHQ-28	16.91 ± 4.82	14.73 ± 5.48	15 ± 4.6
MMSE	29.17 ± 0.79	29.03 ± 1.07	29.21 ± 0.73

Characteristics were not significantly different between treatment groups

*FSH* follicle-stimulating hormone, *GHQ-28* General Health Questionnaire-28, *LMP* time since last menstrual period, *METs* metabolic equivalents, *MMSE* mini-mental state examination, *PA* physical activity

^a^Data on smoking, alcohol use, education, and physical activity were unavailable for 4 participants (*n* = 1, *n* = 2 and *n* = 1 in the low-, medium-, and high-dose groups, respectively) as they failed to complete the health and lifestyle questionnaire

^b^Mean ± SD (all such values with the exception of smoking and education)

**Table 2:** Cognitive function of post-menopausal women at baseline and following 12 weeks’ soy drink intervention

Treatment group	Low (*n* = 35)			Medium (*n* = 37)			High (*n* = 29)		
Cognitive test	Baseline	Week 12	Change from baseline	Baseline	Week 12	Change from baseline	Baseline	Week 12	Change from baseline
RTI	373.66 ± 51.83^a^	367.06 ± 59.34	− 6.60 ± 55.11	345 ± 45.66	354.15 ± 67	9.15 ± 55.07	349.33 ± 61.70	363.99 ± 70.79	14.66 ± 52.64
SSP	5.51 ± 1.06	5.77 ± 1.14	0.25 ± 1.22	5.65 ± 1.18	5.68 ± 1.23	0.03 ± 1.55	5.62 ± 1.18	5.76 ± 0.83	0.14 ± 1.36
SWM	33.86 ± 19.09	29.11 ± 16.95	− 4.74 ± 15.74	28.7 ± 17.59	28.92 ± 17.85	0.22 ± 17.42	30.45 ± 16.04	27.90 ± 16.08	− 2.55 ± 15.07
PRM	2054.25 ± 395.87	2083.14 ± 544.32	28.89 ± 417.47	2138.94 ± 587.38	1903.244 ± 434.09	− 235.7 ± 557.83	1920.65 ± 458.59	1969.56 ± 568.19	48.91 ± 565.60
MTS	2836.70 ± 732.05	2518.54 ± 735.28	− 318.16 ± 585.35	2846.98 ± 796.82	2456.46 ± 606.2	− 390.52 ± 814.11	2694.54 ± 597.66	2535.90 ± 607.23	− 158.64 ± 572.23

No significant differences were observed between the groups for any of the outcomes measured using an ANCOVA to compare post-intervention (week 12) cognitive function with baseline measures as covariates

*RTI* five-choice reaction time measured as reaction time latency (ms), *SSP* spatial span measured as longest sequence length recalled correctly, *SWM* spatial working memory measured as total errors made, *PRM* pattern recognition memory measured as mean correct latency (ms), *MTS* match to sample visual search measured as mean correct reaction time (ms)

^a^Mean ± SD (all such values)

**Table 3:** Menopausal symptoms at baseline and following 12 weeks’ soy drink intervention

Treatment group	Low (*n* = 34)			Medium (*n* = 35)			High (*n* = 27)		
Greene’s Climacteric Scale	Baseline	Week 12	Change from baseline	Baseline	Week 12	Change from baseline	Baseline	Week 12	Change from baseline
Psychological score	18.29 ± 4.00^a^	17.47 ± 4.37	− 0.83 ± 5.10	18.51 ± 4.92	16.40 ± 4.11	− 2.11 ± 4.19	17.02 ± 3.55	15.93 ± 3.59	− 1.10 ± 3.89
Vasomotor score	4.46 ± 1.58	3.97 ± 1.73	− 0.49 ± 1.18	4.50 ± 1.54	3.49 ± 1.27	− 1.01 ± 1.84	3.54 ± 1.22	3.37 ± 1.69	− 0.17 ± 2.02
Somatic score	9.79 ± 1.81	9.29 ± 2.28	− 0.49 ± 2.50	9.71 ± 2.30	9.49 ± 2.13	− 0.23 ± 2.68	9.77 ± 2.64	9.26 ± 3.37	− 0.51 ± 3.52
Total score	34.72 ± 5.37	32.76 ± 6.52	− 1.96 ± 6.83	34.87 ± 7.31	31.29 ± 6.10	− 3.58 ± 6.87	32.52 ± 6.42	30.52 ± 7.91	− 2.00 ± 8.62

No significant differences were observed between the groups for any of the symptoms assessed as determined using a Kruskal–Wallis test

^a^Mean ± SD (all such values)

**Table 4:** Effect of soy drink intervention on vasomotor symptoms stratifying women according to severity of symptoms at baseline

Treatment group	Low (*n* = 34)			*P*^a^	Medium (*n* = 35)			*P*	High (*n* = 27)			*P*
	Baseline	Week 12	Change from baseline		Baseline	Week 12	Change from baseline		Baseline	Week 12	Change from baseline	
Less severe	3.37 ± 0.90^b^	3.05 ± 0.97	− 0.32 ± 1.06	0.290	3.22 ± 0.73	3.28 ± 1.18	0.06 ± 1.21	0.001	2.64 ± 0.50	3.14 ± 2.11	0.50 ± 1.99	0.096
More severe	5.83 ± 1.10	5.13 ± 1.81	− 0.70 ± 1.33^A^		5.85 ± 0.83	3.71 ± 1.36	− 2.15 ± 1.73^B^		4.51 ± 0.99	3.62 ± 1.12	− 0.89 ± 1.88^A^	

^a^Mann–Whitney *U* test comparing change from baseline of those women with more severe vasomotor symptoms at baseline to those with less severe symptoms within groups

^b^Mean ± SD (all such values)

^A,B^Values with different superscript letters across a row are significantly different (one-way ANOVA with quadratic contrast analyses)

Compliance, as assessed via plasma IF concentrations, appeared good (Table 5), albeit some women found it difficult to consume the instructed volume of soy drink in the high-dose group, with seven women failing to complete the intervention for this reason. Blood samples were available for IF analysis for 95 participants at baseline and 87 post-intervention. IF concentrations were not significantly different between groups at baseline. As expected, post-intervention genistein concentration was significantly higher in the medium- (*P* = 0.007) and high-dose (*P* = 0.013) groups in comparison to the low-dose group. Post-intervention daidzein was also significantly higher in the medium- (*P* = 0.006) and high-dose (*P* = 0.029) groups versus the low group. There was no significant difference in post-intervention IF concentrations between the medium- and high-dose groups. Some 28.7% of the cohort was classified as equol producers with *n* = 7, *n* = 9, and *n* = 9 participants classified as equol producers within the low-, medium-, and high-dose groups, respectively. In sub-analysis, cognitive performance was not significantly different according to equol status (Table 6) albeit, within the high-dose group, spatial working memory improved in equol producers (change from baseline − 9.44 ± 15.69, *n* = 9) in comparison to non-producers (2.36 ± 13.24, *n* = 23), though this effect did not reach significance (*P* = 0.066). VMS were significantly lower in equol producers in comparison to non-producers at both baseline (3.67 ± 1.01 vs 4.49 ± 1.60, *P* = 0.022) and post-intervention (2.96 ± 1.04 vs 3.86 ± 1.67, *P* = 0.046).

**Table 5:** IF concentrations at baseline and following 12 weeks’ soy drink intervention

Treatment group	Low		Medium		High	
	Baseline (*n* = 32)	Week 12 (*n* = 32)	Baseline (*n* = 35)	Week 12 (*n* = 32)	Baseline (*n* = 28)	Week 12 (*n* = 23)
Genistein (ng/ml)	10.01 ± 22.07^a^	82.75 ± 124.62^A^	9.83 ± 24.25	168.71 ± 166.81^B^	6.40 ± 9.27	216.71 ± 305.36^B^
Daidzein (ng/ml)	5.03 ± 11.88	20.44 ± 30.82^A^	2.29 ± 4.37	39.20 ± 36.9^B^	2.50 ± 3.34	49.82 ± 66.49^B^
Equol (ng/ml)	1.76 ± 6.01	3.81 ± 8.17^A^	BLD	9.43 ± 19.84^A^	0.42 ± 1.30	11.69 ± 19.23^A^

*BLD* below limit of detection

^a^Mean ± SD (all such values)

^A,B^Values with different superscript letters across a row are significantly different (Kruskal–Wallis with post hoc Mann–Whitney *U* test)

**Table 6:** Effect of soy drink intervention on cognitive performance stratifying women according to equol producer status

Cognitive test	Change from baseline		*P*^b^
	Equol non-producers	Equol producers^a^	
	*n* = 62	*n* = 25	
RTI	6.23 ± 54.98^c^	− 6.23 ± 41.30	0.764
SSP	0.21 ± 1.34	0.12 ± 1.42	0.735
SWM	− 1.82 ± 15.34	− 6.16 ± 16.36	0.245
PRM	− 45.58 ± 533.00	− 62.66 ± 557.10	0.453
MTS	− 363.39 ± 675.51	− 163.99 ± 630.38	0.234

*RTI* five-choice reaction time measured as reaction time latency (ms), *SSP* spatial span measured as the longest sequence length recalled correctly, *SWM* spatial working memory measured as total errors made, *PRM* pattern recognition memory measured as mean correct latency (ms), *MTS* match to sample visual search measured as mean correct reaction time (ms)

^a^Defined as plasma equol concentration > 20 nmol/l (5 µg/l)

^b^Independent samples *t* test or Mann–Whitney *U* test for non-parametric data

^c^Mean ± SD (all such values)

Energy intake, carbohydrate, protein, and total fat intake were not significantly different between the low-, medium-, and high-dose groups at baseline or post-intervention (Table 7). In the high-dose group, protein and total fat intake (expressed as % energy intake) were significantly lower post-intervention in comparison to baseline. Using the Goldberg cut-off technique [60], 12.8% and 15.7% of participants were identified as mis-reporters at baseline and post-intervention, respectively. Of these, three were within the low, two within the medium, and five within the high-dose groups at baseline with six within the low, two within the medium, and three within the high-dose groups post-intervention.

**Table 7:** Dietary intake at baseline and following 12 weeks’ soy drink intervention

Treatment group	Low			Medium			High		
	Baseline (*n* = 27)	Week 12 (*n* = 28)	*P*^a^	Baseline (*n* = 27)	Week 12 (*n* = 21)	*P*	Baseline (*n* = 24)	Week 12 (*n* = 21)	*P*
Energy (Kcal/day)	1532 ± 451.72^b^	1506 ± 526.37	0.715	1623 ± 549.91	1792.62 ± 665.34	0.845	1562 ± 501.51	1731 ± 583.89	0.163
Carbohydrate (g/day)	165.52 ± 47.76	154.21 ± 46.65	0.144	165.67 ± 44.26	162.43 ± 50.24	0.500	173.38 ± 63.37	170.00 ± 54.52	0.472
Protein (g)	74.74 ± 42.49	72.39 ± 24.59	0.726	75.85 ± 33.37	71.90 ± 28.28	0.184	77.21 ± 27.46	70.71 ± 32.31	0.139
Fat (g)	61.07 ± 25.96	59.86 ± 18.31	0.935	59.56 ± 16.74	65.40 ± 23.34	0.887	61.21 ± 23.32	59.00 ± 25.08	0.868
Carbohydrate (% EI)	44.32 ± 9.85	45.74 ± 28.91	0.068	42.52 ± 8.99	37.60 ± 7.82	0.184	44.26 ± 5.31	41.00 ± 11.35	0.744
Protein (% EI)	15.55 ± 2.55	18.28 ± 12.52	0.563	15.27 ± 3.42	15.37 ± 5.84	0.500	15.46 ± 2.13	13.59 ± 3.67	0.018
Fat (% EI)	42.76 ± 17.35	47.89 ± 25.98	0.503	42.11 ± 11.52	38.67 ± 12.85	0.112	46.94 ± 19.53	37.71 ± 13.52	0.022

Nutrient intake did not differ between the groups at either baseline or post-intervention (Kruskal–Wallis test)

*EI* energy intake

^a^Wilcoxon signed-rank test comparing week 12 to baseline within groups

^b^Mean ± SD (all such values)

## Discussion

In the current study, consumption of a soy drink for 12 weeks, providing 35 or 60 mg of IFs/day, had no effect on visual memory, working memory, or attention, in apparently healthy post-menopausal women in comparison to consumption of a low-dose control providing 10 mg IFs/day. We did not observe a significant effect for menopausal symptoms; albeit, sub-analysis identified a potential beneficial effect of soy IFs in women with more severe VMS at baseline. Our findings on cognitive function are in agreement with the previous studies that observed no beneficial effects of isoflavone supplementation on cognition in post-menopausal women [30, 32, 33]. In contrast, others have demonstrated an improvement in frontal lobe function (mental flexibility and planning) [24, 26], long-term episodic memory, sustained attention [24], psychomotor performance [27], verbal/semantic memory [25], and visual memory [29]. Variations in study design make it difficult to draw direct comparisons between the findings of our study and previous work. Mixed results may be reflective of the different cognitive tests used. Although two studies, showing beneficial effects, utilised CANTAB to assess cognitive function [24, 26], neither used the same tests as the current study. Given the wide range of methodologies currently available to assess cognitive function, there is a need to identify a standard method and testing suite to enable a better comparison between studies and reliably inform scientific knowledge in this area. Conflicting results may also be reflective of variation in the dose and duration of isoflavone supplementation used. Three studies that report improvements in cognitive function with soy IF supplementation have used much higher doses and/or have intervened over a longer duration than that used in the current study [25, 27, 29]. Furthermore, age/time since menopause may be a key factor in determining response to soy isoflavones, with Kritz-Silverstein et al. [25] observing significant effects on a test of visumotor tracking and attention in younger (50–59 years) but not older women (60–74 years). Kreijkamp-Kaspers et al. also observed no significant effects using the same test in a cohort of older post-menopausal women, aged 60–75 years [30]. In the longest intervention trial conducted to date, supplementation of 91 mg IFs daily for 2.5 years had no effect on global cognition, executive function, or verbal episodic memory in healthy post-menopausal women albeit, an improvement in a visual memory factor was observed [29]. In contrast to our study, there was a wide age range in this study (45–92 years) with almost half of the cohort > 10 years postmenopause; sub-analysis suggested that such women were less likely to show cognitive improvement and this may, therefore, have influenced the null findings in this study. Finally, differences in the makeup of the supplement used, in terms of the individual isoflavone constituents, may account for the observed mixed findings on cognition. Genistein and daidzein have distinct biological effects, e.g., genistein is a potent inhibitor of protein tyrosine kinase (PTK) [61], whereas daidzein is not a PTK inhibitor [62]. Genistein is now thought more promising as a treatment for Alzheimer’s disease [63–65].

To our knowledge, only one other study has investigated the effects of IFs on post-menopausal cognition in the form of a soy drink [31]. In this study, consumption of IFs via a drink (72 mg/day) or a supplement (70 mg/day) over 16 weeks did not improve short-term memory, long-term memory, working memory, or selective attention as assessed using tests similar to those used in our study. The soy milk group showed a poorer performance in verbal working memory in comparison to the supplement and control groups; albeit this study was subject to limitations including subjective compliance, lack of power, and lack of controls, owing to the quasi-experimental design.

In agreement with the previous literature, 28.7% of our study cohort were equol producers [38–40]. Limited evidence suggests that the ability to produce equol may confer beneficial effects on cognitive function following soy intake [66, 67], potentially via increased cerebral blood flow [68]. We did not observe any significant differences in cognitive performance following intervention between equol producers and non-producers, although, within the high-dose group, improvements in spatial working memory in producers versus non-producers were approaching significance. Our findings support those of Henderson et al. [29] who observed a nonsignificant trend towards improved global cognition in consistent equol producers. Similar to Henderson’s study, the sample size in the current study was likely too small to adequately investigate the role of equol in cognitive performance and further research in this area is warranted.

In agreement with our findings, previous studies, including 2 that used the same scale as that in the current study [24, 26], have reported no effect of IF supplementation on menopausal symptoms [24, 26, 32]. Basaria et al. observed an improvement in menopausal symptoms using the menopause-specific quality-of-life questionnaire; however, the dose used in this study was very high (160 mg/day) [33]. A recent meta-analysis has demonstrated that soy IF supplementation can significantly reduce hot flush frequency and severity in comparison to placebo, with supplements containing > 18.8 mg of genistein being most effective [12]. In support of these findings, our study has shown that women with more severe VMS at baseline showed a significant improvement in symptoms following consumption of 35 mg IFs/day (providing ~ 18 mg genistein) in comparison to those consuming 10 mg/day. This observation was not replicated in the high-dose group, possibly due to the sample size, and thus, further studies are required to confirm these findings. Our findings in the medium group are in agreement with an open-label crossover study that demonstrated a commercial soy drink (ViveSoy^®^), providing ~ 50 mg IFs per day for 12 weeks, improved climacteric symptoms including hot flushes in peri/post-menopausal women [69]. Previous research on the effects of soy foods on menopausal symptoms is limited [70] and our findings warrant further study in this area. In support of the role of S-equol in the alleviation of VMS [41], equol producers in the current study had significantly lower VMS scores throughout in comparison to non-equol producers.

Strengths of this study include the use of CANTAB, a well-validated method in digital cognitive assessment. CANTAB is a sensitive method to detect changes in cognitive domains, including spatial memory, executive function, and information processing, in response to nutrition intervention trials, including soy isoflavone trials [71]. Despite being a more sensitive measure, one limitation of this method, highlighted within a recent International Life Sciences Institute (ILSI) review [71], is the speed/accuracy trade-offs associated with the more complex information processing tasks available within computerized test batteries as well as training/practice effects in comparison to simple information processing tasks. In the current study, cognitive assessment was conducted 12 weeks apart, and thus, training/practice effects would be negligible. Cognitive assessment was strictly controlled with participants consuming the same standard caffeine-free breakfast prior to testing at both timepoints. Time since menopause was kept within a defined limit of < 7 years and compliance was closely monitored. The main limitations of the study were the absence of a placebo control and the non-blinded study design. Nonetheless, the low-dose control drink provided only 10 mg IFs/day, an amount well below that typically consumed in Asian populations (~ 25–50 mg/day) [72] and well below the lowest dose previously used in intervention studies on cognitive function in post-menopausal women (60 mg/day) [24, 26, 27]. Given that we used a commercially available soy drink in the current intervention study, we were limited with regard to the dose of IFs that could be tested. Nonetheless, it is important to investigate the effects of soy foods in addition to IF supplements as consumers may gain additional nutritional benefits from consuming soy food as part of a healthy balanced diet [73]. Furthermore, IF pharmacokinetics are similar following ingestion of soy foods and IF supplements [74]. We did not reach our recruitment target of 41 participants/group, largely due to participants not meeting the stringent inclusion criteria (of note was the substantial number of post-menopausal women currently on antidepressants); however, the study was still adequately powered (80% power) to detect significant effects. Reference ranges are not provided for the CANTAB tests used for healthy populations; however, our data concur with a previous study in healthy older women [75].

Our participants were apparently healthy and relatively early post-menopausal (< 7 years; mean 3.05 ± 1.78 years post-menopausal) and our findings are, therefore, not representative of all post-menopausal women. Women > 7 years post-menopausal are less likely to be prone to fluctuating hormone levels and associated menopausal symptoms [76]. Furthermore, our findings cannot be extrapolated to post-menopausal women suffering mild cognitive impairment; further research in this area is needed.

In conclusion, a commercially available soy drink had no effect on cognitive function in post-menopausal women. Consumption of ~ 350 ml/day (providing 35 mg IFs) for 12 weeks significantly reduced VMS in those women with more severe symptoms at baseline, a finding with potential clinical relevance that warrants further research given the high prevalence of VMS in western women [77, 78].
